# Epidemiology and diagnostic accuracy of *Clostridium perfringens* toxins in the intestinal contents of camels, sheep, and cattle: a cross-sectional study in Dakahlia governorate, Egypt

**DOI:** 10.1007/s11250-024-04034-7

**Published:** 2024-07-13

**Authors:** Ali Wahdan, Mahmoud M. Elhaig

**Affiliations:** 1 Bacteriology, Immunology, and Mycology Department, Faculty of Veterinary Medicine, Suez Canal University, Ismailia, 41522 Egypt; 2 Department of Animal Medicine (Infectious Diseases), Faculty of Veterinary Medicine, Suez Canal University, Ismailia, 41522 Egypt

**Keywords:** *C. perfringens*, Toxinotyping, Diagnostic accuracy, Camels, Sheep, Cattle

## Abstract

This study aimed to establish an accurate epidemiological surveillance tool for the detection of different *C. perfringens* types from 76 diseased and 34 healthy animals in Dakhalia Governorate, Egypt. A total of 110 intestinal content samples were randomly collected from camels, sheep, and cattle. *C. perfringens* was isolated and biochemically identified by the VITEK2 system. Toxinotyping and genotyping of *C. perfringens* isolates were specified by a multiscreen ELISA and real-time qPCR (rt-qPCR). The occurrence of *C. perfringens* was highest among camels (20% in healthy and 25% in diseased) and was lowest in cattle (23.1% and 14.7%). The *cpa* toxin was detected in all isolates by rt-qPCR and in 7 isolates by ELISA, *ext* toxin was detected in 7 isolates by rt-qPCR and in 6 isolates by ELISA, and c*pb* toxin was detected in 2 isolates by both rt-qPCR and ELISA. Four types of *C. perfringens* were identified by rt-qPCR, type A (65.2%), B (4.3%), C (4.3%), and D (26.1%), and three types by ELISA, type D (17.4%), A (8.7%) and C (4.3%). Our study indicated the prevalence of infection in Dakahlia by *C. perfringens* type A and D, particularly camels, and recommends adopting an appropriate vaccination strategy among the studied animals.

## Introduction

Enterotoxemia affecting all livestock causing severe financial losses due to increase the mortality rate. All types of *C. perfringens* were incriminated for causing the disease(Baums et al. 2004; Greco et al. 2005). *C. perfringens* type A is commonly causing enterotoxaemia and gas gangrene. Type B is associated with hemorrhagic enteritis, and dysentery in lambs. Type C is accompanied by hemorrhagic necrotic enteritis in different species (Kadra et al. 1999). Type D causes enterotoxaemia and pulpy kidney in ovine (Alsaab et al. 2021; Uzal et al. 2010; Uzal and Songer 2008).

*Clostridium perfringens* is gram-positive, anaerobic spore-forming bacilli. It generally presents in intestine of different livestock and humans as well as in the soil (Kiu and Hall 2018; McClane et al. 2006). The disease is caused by different factors, mainly abrupt changes in diet which give the chance for *C. perfringens* to activate and emit variable types of toxins. These toxins either major or minor have a substantial role in the appearance the signs of enterotoxaemia with some irritability and nervous signs on the host after absorption from the intestine (Aktories et al. 2018; Kiu and Hall 2018).

Previously, typing of *C. perfringens* is based on a toxin neutralization test, which have disadvantages as it laborious and tedious. Serotyping of clostridial toxins by ELISA or genotyping by DNA recognition of different sequences for *cpa, cpb, etx*, and *iap* which encode alpha, beta, epsilon, and iota toxins, respectively (Alsaab et al. 2021; Naylor et al. 1997) are the commonly accurate methods nowadays for typing of *C. perfringens*.

Rapid detection of enterotoxaemia in different livestock is a crucial step to prevent the dissemination and spread of the diseases, beside the control strategy of the disease in the farm (Greco et al. 2005; McClane et al. 2006). Traditional cultivation is the backbone of the diagnosis of *C. perfringens* but it takes more time, has low sensitivity, and did not have the ability to differentiate between different types of *C. perfringens* (Eckstein et al. 2022; Gkiourtzidis et al. 2001).

In surveillance and monitoring programs of *C. perfringens* diagnosis from intestinal contents, molecular screening is the first selected technique either multiplex or real-time qPCR (rt-qPCR) as it rapid, accurate, and easily differentiate between different types (Alsaab et al. 2021; Baums et al. 2004; Fayez et al. 2021). Serological assay by ELISA is used in the field for its high sensitivity (Naylor et al. 1997), but accuracy for detection of *C. perfringens* toxins by ELISA and other techniques not well be elucidated. So, this study aimed to estimate the diagnostic accuracy of *C. perfringens* toxins isolated from the intestinal contents of camels, sheep, and cattle during epidemiological surveillance in Dakahlia governorate, Egypt.

## Materials and methods

### Sample collection

**Fig. 1:**
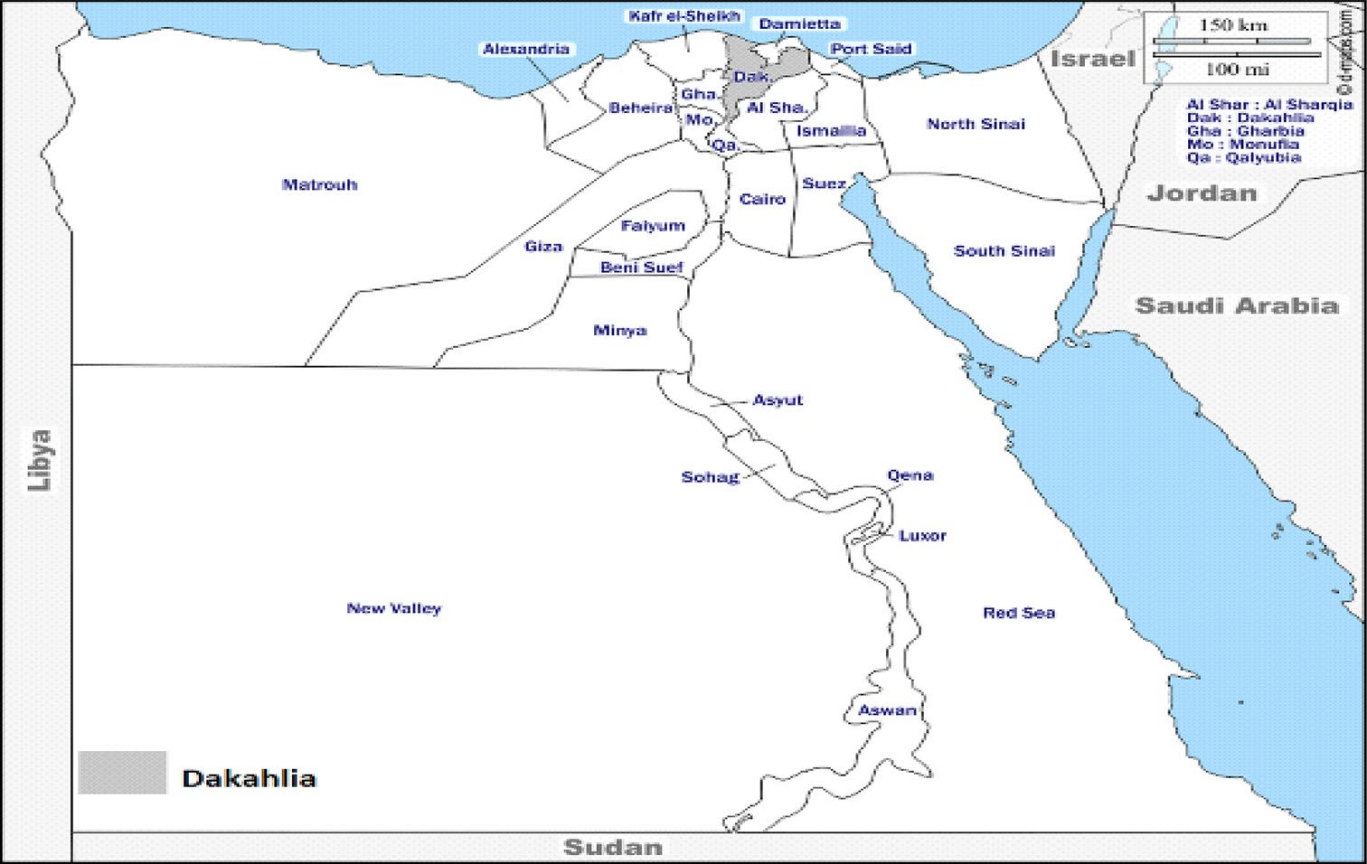
Location (Dakahlia Governorate, Egypt) of sampling under the study. Map was conducted using d-maps

The study was conducted during the year 2021, to examine the presence of *C. perfringens* among camels, sheep, and cattle raised in the Dakahlia governorate, Egypt (Fig. 1). A total of 110 intestinal content samples from slaughterhouse were randomly collected from 76 diseased animals (20 camels, 43 sheep, and 13 cattle) and 34 healthy animals (10 camels, 14 sheep, and 10 cattle) immediately after slaughtering, placed in labelled sterile containers, and transported on ice to the laboratory for preparation and bacteriological investigations. Data for each sample including health status and animal species were recorded (Table 1).

**Table 1:** Prevalence of *C. perfringens* in diseased and healthy camels, sheep and cattle using bacterial culture and ViteK2 methods

Variable	No. of samples	Isolation	Vitek2	Prevalence %	*P*-value
Diseased animals *					
Camels	20	8	5	25	0.9 ^NS^
Sheep	43	19	10	23.3	
Cattle	13	6	3	23.1	
Subtotal (*n* = 76)		33	18	23.7	
Healthy animals					
Camels	10	3	2	20	0.8 ^NS^
Sheep	14	4	2	14.3	
Cattle	10	3	1	10	
Subtotal (*n* = 34)		10	5	14.7	
Total (*n* = 110)		43	23	20.9	

NS, the result is not significant at *P* > 0.05; *, *P* = 0.3 regarding the isolation of ***C. perfringens*** strains from diseased and healthy animals. *P* = 0.8 (diseased camels vs. healthy camels), *P* = 0.5 (Diseased sheep vs. healthy sheep), *P* = 0.4 (diseased cattle vs. healthy cattle)

### Characterization of *C. perfringens*

Samples were gathered by scrubbing the inner wall of the collected intestine by a surgical blade, then the contents were streaked on 5–10% Sheep blood Agar (Oxoid, UK) by cotton swabs, incubated anaerobically at 37 °C for 2 days as described by (Alsaab et al. 2021). Growth characters of the recovered colonies, and morphological appearance by gram stain were examined (McVey et al. 2013). The separated colonies were subjected to biochemical characterization.

### Biochemical confirmation of isolates

The identified colonies were confirmed by VITEK 2 system (bioMérieux, France) using ANC cards (Pincus 2006). The colonies were dispersed in 3 ml VITEK2 saline solution. The turbidity was measured by a Densicheck VITEK 2 at 2.70–3.30 according to the kit instructions. The measured tubes and cards were subjected to VITEK2 apparatus. All data of samples were added to VITEK2 software and saved. The final results and probability of identification were obtained in a result sheet.

### ELISA typing of *C. perfringens* toxins

A sandwich Enterotoxaemia ELISA kit (Multiscreen Ag ELISA, Bio-X Diagnostics, Belgium) was applied for detection of *C. perfringens* and alpha, beta, epsilon toxins in intestinal contents. The liquid intestinal contents from scrubbing were mixed volume per volume with diluted concentrated buffer 5 × (1: 4) in distilled water. One hundred µl of each diluted samples and control antigen were transferred into the wells of an ELISA plate and proceeding according to the directions of the kit’s manufacture. Control antigen was provided by the kit. The test is considered valid when alpha toxin OD > 1.12, Beta toxin OD › 1.108, Epsilon toxin OD > 0.910, *C. perfringens* OD > 1.231. The results are calculated according to the following formula:

1 $${\rm{Value = }}{{{\rm{Delta}}\,{\rm{OD}}{\,_{{\rm{sample}}}}{\rm{ \times 100}}} \over {{\rm{Delta}}\,{\rm{OD}}{\,_{{\rm{positive}}}}}}$$

### Molecular typing of *C. perfringens* toxin

DNA was extracted directly from intestinal contents using QIAamp® DNA extraction kit, (QIAGEN Cat. No.51304), following the protocol of the producer. The PCR protocol was conducted by using a set of primers and probes obtained from Metabion (Germany). Primers and probes details are seen in Supplementary Table. PCR reaction volume (20 µl) consists of 2 µl Light cycler DNA master mix 10X, 3 µl primers and probe of each target sequence, 10 µl PCR water, and 5 µl extracted DNA (Lee et al. 2011). DNA free water was used as negative control, While *C. perfringens* ATCC 19574 was used as positive control. The LightCycler® Roche program was adjusted at (Quantification mode) and the reading of signals was collected at extension phase (Gurjar et al. 2008). The program started by primary denaturation at 95 °C for10 min, followed by denaturation at 95 °C for 30 s, annealing and extension at 55 °C for 1 min (Gurjar et al. 2008).

### Statistical analysis

The Chi-square test was used to evaluate the association between the prevalence of *C. perfringens* and various variables. In addition, the kappa agreement between the results of ELISA and PCR results for toxin detection was calculated using online statistical tools (http://vassarstats.net).

## Results

### Isolation and prevalence of *C. perfringens*

As shown in Table 1, a total of 110 intestinal contents from enterotoxemic and healthy camels, sheep and cattle were cultured and 43 isolates of *C. perfringenes* were detected. Of the 43 isolates, 23 (20.9%) isolates were confirmed by Viteke2 (18 were from diseased animals and 5 were from healthy). Regarding the effect of animal species on the prevalence of *C. perfringens*. There were differences in the *C. perfringens* prevalence in the animals. The prevalence was higher in diseased and healthy camels by 25% and 20%, respectively, than in sheep and cattle, but the difference was statistically non-significant (*P* > 0.05), Table 1. Likewise, the prevalence was non-significant (*P* > 0.05) higher in diseased animals compared to healthy animals.

### Typing of *C. perfringens* isolates by rt-qPCR

**Fig. 2:**
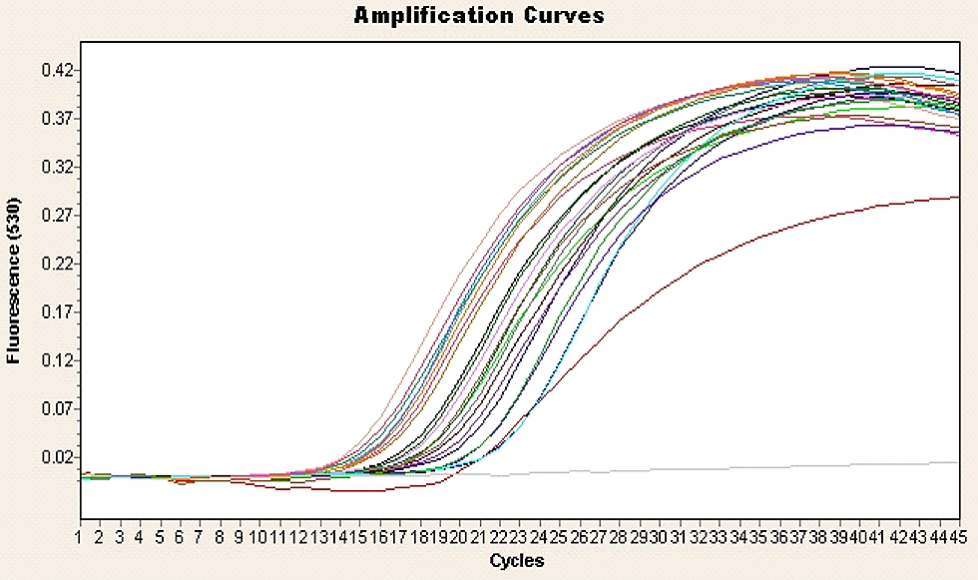
Amplification plot of *cpa* toxin gene of *C. perfingens*

**Fig. 3:**
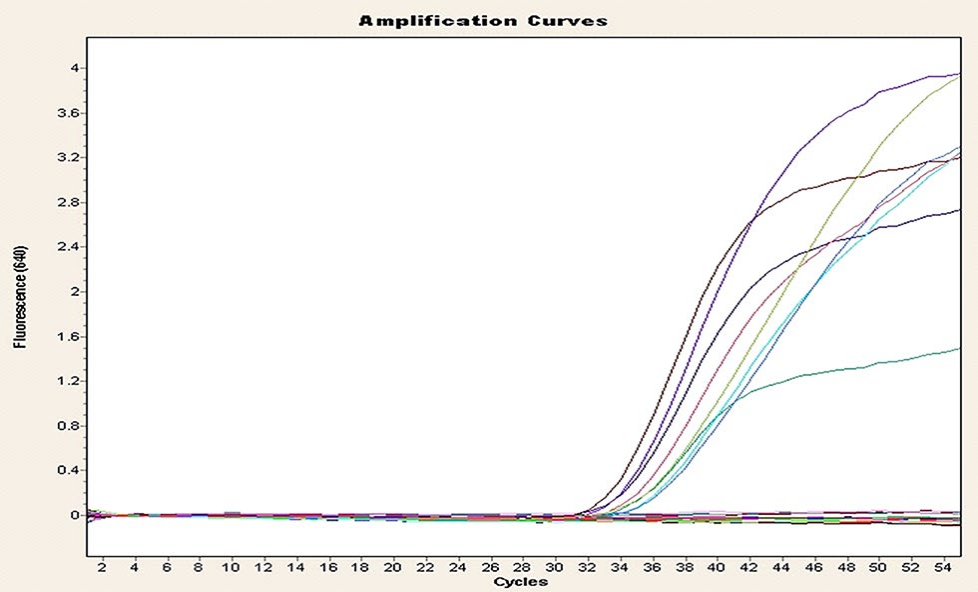
Amplification plot of *etx* toxin gene of *C. perfingens*

**Fig. 4:**
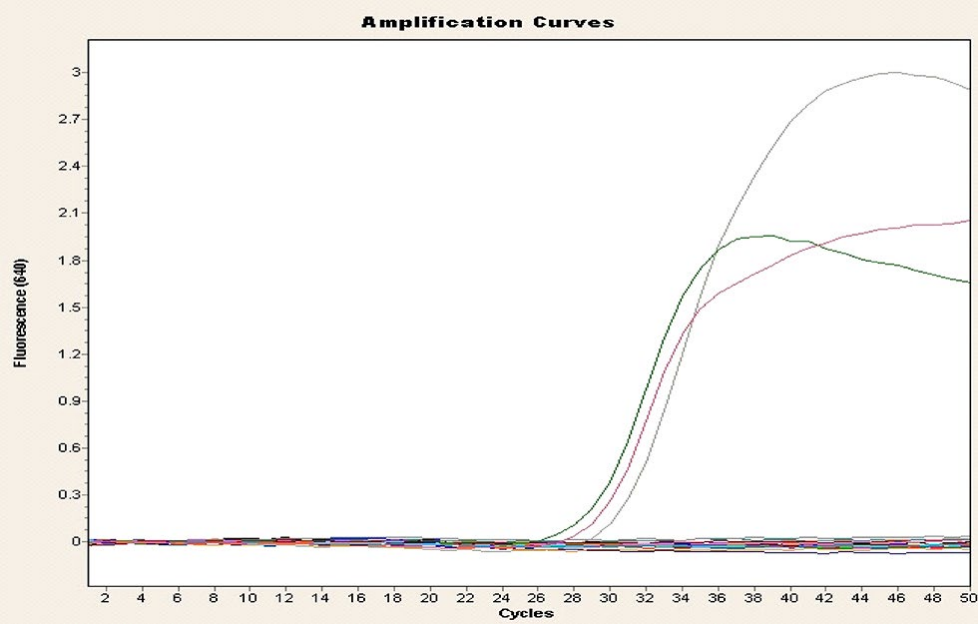
Amplification plot of *cpb* toxin gene of *C. perfingens*

Toxin genotyping of the 23 isolates (Table 2; Figs. 2, 3 and 4) by rt-qPCR targeting *cpa* (*alpha*), *cpb* (*beta*) and *ext* (*epsilon*) toxin genes revealed that *cpa* gene was found in all isolates, *cpb* in 2 isolates, and *etX* in 7 isolates. The overall numbers of toxin genes in the isolates identified among different animal species are shown in Table 2. No isolates from healthy camels, sheep and cattle were positive for *cpb* by rt-qPCR, while no isolates from diseased cattle were positive for the *cpb* and *ext* genes. By analyzing the rt-qPCR results there was a significant difference (*P* < 0.05) with respect to the type of *C. perfringens* among the animals examined. *C. perfringens* type A had the highest prevalence (65.2%), followed by type D (26.1%) and types B and C appeared with the lowest prevalence of 4.3% each, Table 3. Further, there was a significant higher toxins prevalence (*P* < 0.05) in isolates from diseased animals compared to healthy animals. The overall distribution of *C. perfringens* types between diseased and healthy animals is shown in Table 3.

**Table 2:** Distribution of toxin genes in *C. perfringens* strains from camels, sheep and cattle using ELISA and rt-qPCR

Animals	Healthy status	ELISA			Real time PCR		
		cpa (%)	cpb (%)	etx (%)	cpa (%)	cpb (%)	etx (%)
Camels	Diseased (*n* = 5)	0	1 (20)	0	5 (100)	1 (20)	0
	Apparent healthy (*n* = 2)	1 (50)	0	1 (50)	2 (100)	0	1 (50)
Sheep	Diseased (*n* = 10)	4 (40)	0	3 (30)	10 (100)	0	3 (30)
	Apparent healthy (*n* = 2)	1 (50)	0	2 (100)	2 (100)	0	1 (50)
Cattle	Diseased (*n* = 3)	1 (33.3)	1 (33.3)	0	3 (100)	1 (33.3)	1 (33.3)
	Apparent healthy (*n* = 1)	0	0	0	1 (100)	0	1 (100)
**Total** (*n* = 23)		**7 (30.4%)**	**2 (8.7%)**	**6 (26.1)**	**23 (100%)**	**2 (8.7%)**	**7 (30.4%)**

**Table 3:** Prevalence of *C. perfringens* types isolated from diseased (*n* = 18) and healthy animals (*n* = 18)

Animals	Type A		Type B		Type C		Type D		*P*-value
	cpA		cpA + cpB + etX		cpA + cpB		cpA + etX		
	*N*	%	*n*	%	*n*	%	*n*	%	
Diseased camels	4	26.7	0	0	1	100	0	0	-
Diseased Sheep	7	46.7	0	0	0	0	3	50	-
Diseased cattle	2	13.3	1	100	0	0	0	0	-
Subtotal (*n* = 18)	13	86.7	1	6.7	1	6.7	3	20	< 0.00001*
Healthy Camels	1	6.7	0	0	0	0	1	16.7	-
Healthy Sheep	1	6.7	0	0	0	0	1	16.7	-
Healthy cattle	0	0	0	0	0	0	1	16.7	-
Subtotal (*n* = 5)	2	13.3	0	-	0	-	3	20	0.01*
Total (*n* = 23)	15	65.2	1	4.3	1	4.3	6	26.1	< 0.00001*

*, The result is significant at *P* < 0.05

### ELISA-typing of *C. perfringens* isolates

The results of ELISA showed that *cpa* toxin was the highest (30.4%, *n* = 7; 5 from diseased sheep and cattle, 2 from healthy camels and sheep), followed by *ext* toxin (26.1%, *n* = 6; 3 from diseased sheep, 3 from healthy camels and sheep), and *cpb* toxin (8.7%, *n* = 2; one from both diseased camel and cattle). No toxins were detected in healthy cattle. Further details about distribution of toxins are listed in Table 2. By analyzing the ELISA results, *C. perfringens* type D was the highest prevalent (4/23, 17.4%), followed by type A (2/23, 8.7%) and type C was the least (1/23, 4.3%). The ELISA test could not detect type B, since the only toxin identified in diseased camels was *cpa* toxin.

### Test agreement

More toxins were identified by rt-qPCR than by ELISA for *cpa* toxin (100% by rt-qPCR vs. 30.4% by ELISA) and *ext* toxin (30.4 by rt-qPCR vs. 26.1 by ELISA), however, *cpb* was equally detected by both assays (8.7% each). By comparing results of ELISA and rt-qPCR (Table 4), regarding *cpa* detection, no agreement was calculated because all samples were rt-qPCR positive giving the superiority of rt-qPCR over ELISA, which detected 7 samples harbored *cpa* toxin. Concerning *cpb*, there was perfect agreement (K = 1) between both assays, but the agreement was substantial (K = 0.7, 95% CI 0.34–1) when *ext* toxin was considered. Moreover, when comparing the overall positivity rate in both tests, which depends on whether any toxin was detected in a sample considered positive, there was slight agreement (K = 0.1) between the rt-qPCR and ELISA results.

**Table 4:** The agreement between results of rt-qPCR and ELISA in toxin detection among 23 *C. perfringens* isolates

Variable		rt-qPCR		Total	K value	95% CI
		Pos	Neg			
ElISA-*cpa*	Pos	7	0	7	ND	-
	Neg	16	0	16		
	Total	23	0	23		
ELISA-*cpb*	Pos	2	0	2	1	0–1
	Neg	0	21	21		
	Total	2	21	23		
ELISA-*ext*	Pos	5	1	6	0.7	0.34–1
	Neg	2	15	17		
	Total	7	16	23		
ELISA	Pos	9	0	9	0.1	0–0.4
	Neg	13	1	14		
	Total	22	1	23		

## Discussion

*Clostridium* perfringens is a significant bacterium of the gut microbiota, and some strains are known to cause diseases in humans and animals such as myonecrosis, food poisoning, enterotoxemia, and enteritis. Toxins cause the enteric diseases and consequent economic losses, especially in livestock (Greco et al. 2005). There may be geographic divergences in the predominant bacterial species and the species may also vary depending on the species of the animals of the region (Yoo et al. 1997).

Recently, there are several reports in Egypt about the increased detection of *C. perfringens*, mostly in cattle, buffaloes, sheep, goats, and chicken (Bendary et al. 2022; Moustafa et al. 2022; Selim et al. 2017). However, this study is one of the very few works in Egypt evaluating the toxins, genotypes and prevalence of *C. perfringens* in camels (Ahmed et al. 2022; E Mohamed et al. 2010). The exact origin of *C. perfringens* isolates derived from Egyptian animals particularly camels are controversial and pose a risk factor due to the instability of the camel population caused by increased import and demand. From this it can be conclude that the early detection of *C. perfringens* can lead to reduced losses and rapid application of control in affected areas.

Our findings indicated that 23 isolates of *C. perfringens* (20.9%) were obtained from all animals examined (camels, sheep, and cattle), and the 65.2%, 26.1%, 4.3%, and 4.3% of the isolates were type A, D, B, and C, respectively.

Using the bacteriological methods, *C. perfringens* was detected in 20.9% of 110 intestinal content samples in the Dakahlia governorate, Egypt. This result is lower than previous studies from Egypt that reported a prevalence of 51.5–62.14% in lambs from Menofia and Qalyubia governorates (Moustafa et al. 2022), 77.1% in calves from Qalyubia governorate (Selim et al. 2017), and 55.81% in cattle, sheep and goats from Cairo and Giza governorates (Hamza et al. 2018). In Saudi Arabia, the prevalence was 56.3% in camels (Fayez et al. 2021) and 27.2% in livestock (cattle, goats, sheep and camels) (Omer et al. 2020), and also in Khuzestan, the prevalence was 32.1% in sheep and goats (Rahaman et al. 2013).

In contrast to our results, in Egypt and other countries, lower rates of *C. perfringens* were reported than reported in this study. In Egypt, the prevalence was 4% in buffalo and 4.48% in cattle (Osman et al. 2009), but the studied area was not specified, and 4–15.2% in chicken and various animals from Sharkia and Port Said governorates (Ahmed et al. 2022; Bendary et al. 2022). However, in another countries, the prevalence rate was lower than reported here. In Saudi Arabia, it was 14% for camel meat (Fayez et al. 2021) and15% for diarrheal sheep (Alsaab et al. 2021), and 10.76% in India for multi-species (Anju et al. 2021). The variable rate of *C. perfringens* can be attributed to differences in the hygienic status of the studied groups, the laboratory tests used, the animal species and the geographical location (Khan et al. 2015; Omer et al. 2020).

*C. perfringens* was isolated from all studied animal species (camels, sheep, and cattle) at varying rates, but a non-significant difference (*P* > 0.05) was reported. *C. perfringens* was highest in camels (20% and 25%), followed by sheep (14.3 and 23.3%) and lowest in cattle (10% and 23.1%) in healthy and diseased cases, respectively. A study in Egypt revealed that the detection of *C. perfringens* in apparently healthy animals was highest in sheep (65.45%), followed by goats (58%), buffalo (55%) and cattle (47.1%), with no significant difference (*P* > 0.05) (Hamza et al. 2018). However, a study from Saudi Arabia found that *C. perfringens* was most frequently detected in cattle (64.3%), followed by goats (29.9), camels (21.5%), and sheep (21.4%), with significant difference (*P* < 0.05) (Omer et al. 2020). The pattern of occurrence of *C. perfringens* in the present and previous studies is consistent with the growing concern about enterotoxaemia that has been focused in Egypt and elsewhere and with established certainty that *C. perfringens* is widely distributed in nature and commonly found in the intestine of animals as previously reported (Bokaeian et al. 2015; Hamza et al. 2018).

Although **i**n the current study, camels have the high rate of *C. perfringens*, few studies have documented *C. perfringens* infection in camels; for example, two studies from Egypt (Ahmed et al. 2022; E Mohamed et al. 2010) reported that the infection rate of camels reached 18 and 26.7%, as well as two studies from Saudi Arabia (Fayez et al. 2021; Omer et al. 2020) reported an infection rate of 21.5 and 56.3%. This could be attributed to the early *clostridium* colonization of the gut in the form of acute and subacute infections (SANOUSI and Gameel 1993) or the existence of other infections, such as trypanosomiasis, which in turn lowered the immunity of camels and salmonellosis, which in turn damaged the gut mucosa and therefore causing an increase in the rate of the *C. perfringens* isolation (Wernery et al. 1991). Also, in accordance to (Fayez et al. 2021) mixing camels and small ruminants provides a possible source of *C. perfringens* especially types B, C, and D for camels. Moreover, according to (Seifert 1996) a diet high in carbohydrates and protein and a change in climate or habitat can create optimal conditions for the propagation of perfringens in animals, like the same in this study where camel numbers have recently increased in Egypt mostly in the Delta region (Alfaleh and Elhaig 2023) for the purpose of fattening with a diet rich in protein and carbohydrates. The relatively high percentage of *C. perfringens* in camels in this study is worthwhile and of clinical and epidemiological importance and raises significant queries about the origin, distribution, and reservoir effect of *C. perfringens* in the Egyptian environment, particularly rural areas.

Our findings revealed that all identified *C. perfringens* types produce *cpa* toxin by rt-qPCR and 7 isolates were *cpa*-positive by ELISA; however, the frequency of *cpa* toxin is higher in diseased and healthy animals than the other toxins, a finding is endorsed by previous studies on *C. perfringens* in animals and humans (Ahmed et al. 2022; Aschfalk and Müller 2002; Canard et al. 1992) .

In this study, analysis of PCR results revealed that *C. perfringens* type A was the most prevalent (65.2%), followed by type D (26.1%) and types B and C were the least prevalent (4.3% each). A study in Italy reported that by PCR, 93% of the *C. perfringens* isolates of animal origin were type A, 3% were type D, and 2.5% were type F (Forti et al. 2020). Also, in Saudi Arabia, genotyping by PCR showed that 75.2%, 13.7%, 6.9% and 4.2% of *C. perfringens* from camels were type A, C, D, and B, respectively (Fayez et al. 2021). In a study conducted in Turkey to investigate the types of *C. perfringens* isolates in lambs by PCR found that type A was the most (76.92%), followed by type D (15.38%), type C (7.69%) and types B and E were not detected (HADİMLİ et al. 2012).

Moreover, a study conducted in Egypt on lambs by ELISA reported that 43.68% of *C. perfringens* isolates were type A, 33.98% were type B and 22.33% were type D, whereas type C and E were not found (Moustafa et al. 2022). Also, a study conducted in Egypt on camels and humans through PCR reported that the frequency of *C. perfringens* types A, B, C, D was 65.0%, 10.0%, 2.5%, 2.5%, respectively, whereas type E was not found (E Mohamed et al. 2010). The difference in the types of *C. perfringens* between the current study and the previous studies may be attributed to variance in geography and management, laboratory techniques used, and feeding quality (Yadav et al. 2017).

Toxins typing by ELISA and rt-qPCR revealed that type D (17.4%) was the most prevalent by ELISA and type A by rt-qPCR in both diseased and apparent healthy animals. A previous study in Iran showed that *C. perfringens* was isolated from healthy and enterotoxemic sheep and type A was the predominant by rt-qPCR in both groups and type D was the predominant by ELISA in the clinic group (Hayati and Tahamtan 2021). Another study in Turkey found that the *C. perfringens* type A was the dominant in enterotoxemic sheep by both ELISA and PCR (HADİMLİ et al. 2012).

Moreover, the results of ELISA and rt-qPCR were compared, and rt-qPCR was superior to ELISA in detecting *cpa* and *ext* toxins and they were compatible in detecting *cpb* toxin. In Denmark, testing of 37 mixed cultures of *C. perfringens* of piglet intestinal mucosa by PCR and ELISA concluded that PCR was more effective than ELISA in detecting *cpb* toxin (Møller and Ahrens 1996).

Contrast to rt-qPCR, ELISA showed limited viability in detection of *cpa* toxin and also failed to classify type B of *C. perfringens* although the simplicity and rapid of ELISA for detection and distribution of *C. perfringens* toxins especially with levels detectable during sampling (Waggett et al. 2010).

## Conclusions

This study supports the growing concern about enterotoxaemia that has been focused elsewhere. The results show that *C. perfringens*, especially with the type A, and D is prevalent in camels, sheep and cattle from Dakahlia, Egypt, both clinically and apparently healthy, with an increased prevalence of type A in intestinal content of clinical cases. The rt-qPCR method appears robust in screening and genotyping of *C. perfringens* and tracking its spread in animals. The lack of genome sequencing in the current study prevents us from determining the origin and increased prevalence of *C. perfringens*. Therefore, further studies in this point must be continued to provide a strong plane for the improve control protocols for *C. perfringens*.

## Data Availability

Data will be available upon request.
